# Supplementary figures and images for: Extracellular proteolysis of tandemly duplicated pheromone propeptides affords additional complexity to bacterial quorum sensing
Source: PLoS Biol. 2024 Aug 13;22(8):e3002744. doi: 10.1371/journal.pbio.3002744 (PMC11343458; doi:10.1371/journal.pbio.3002744)

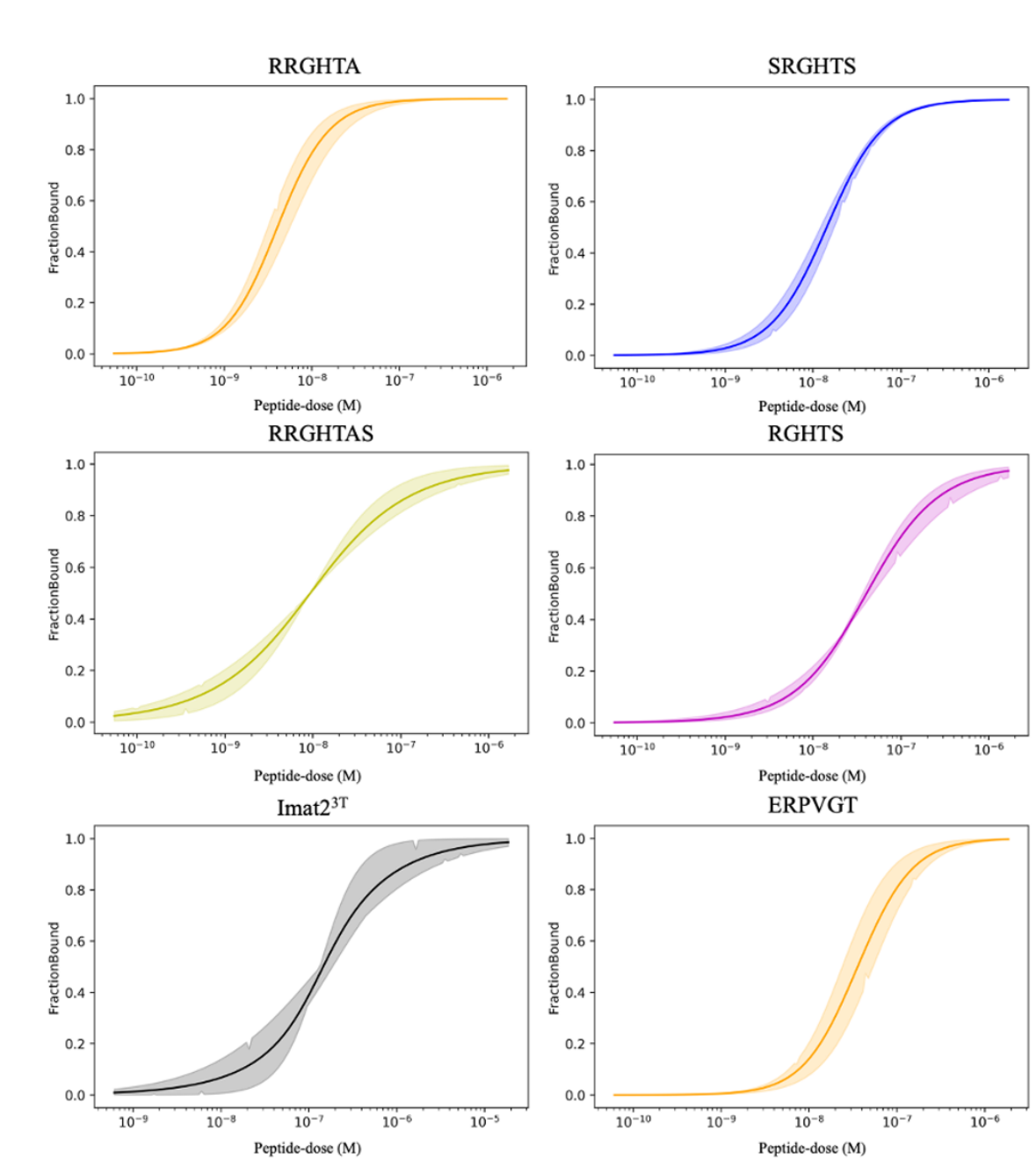

Supplement: S1 Fig — Dose-response slopes for the interaction between Rap3T and pheromone variants (RRGHTA, SRGHTS, RRGHTAS, RGHTS, and Imat13T) and Rap105 with pheromone ERPVGT. Notice the sigmoidal and non-hyperbolic curve, indicating a cooperative effect on peptide binding. (TIFF) [file pbio.3002744.s001.tiff]

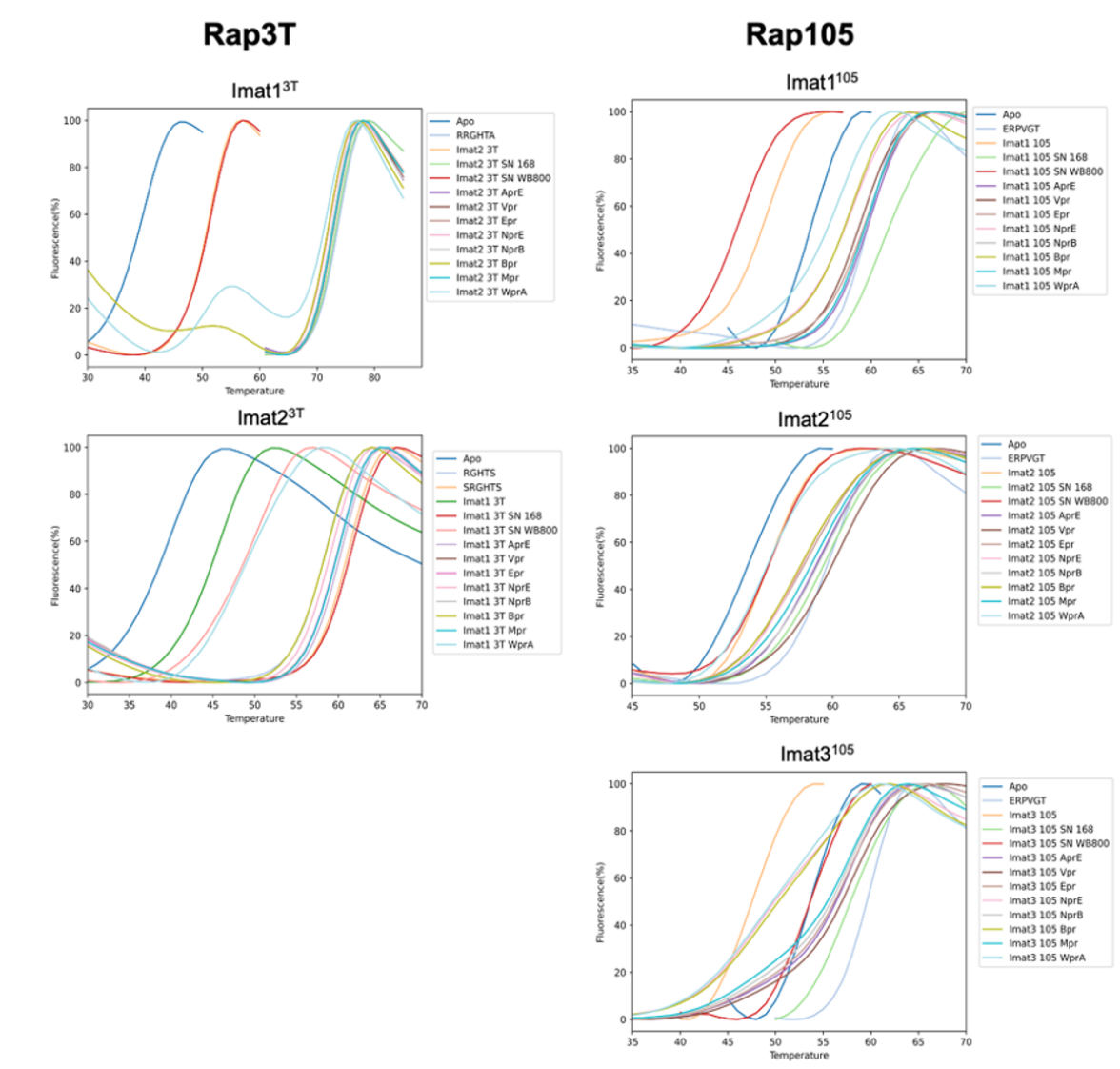

Supplement: S2 Fig — Slopes of representative thermal shift assays with supernatants from B. subtilis 168, WB800, individual protease mutant strains and pheromone controls with Rap3T (left) and Rap105 (right) for maturation of peptides Imat13T(Rap3T), Imat23T(Rap3T), Imat1105 (Rap105), Imat2105 (Rap105), and Imat3105 (Rap105). (TIFF) [file pbio.3002744.s002.tiff]

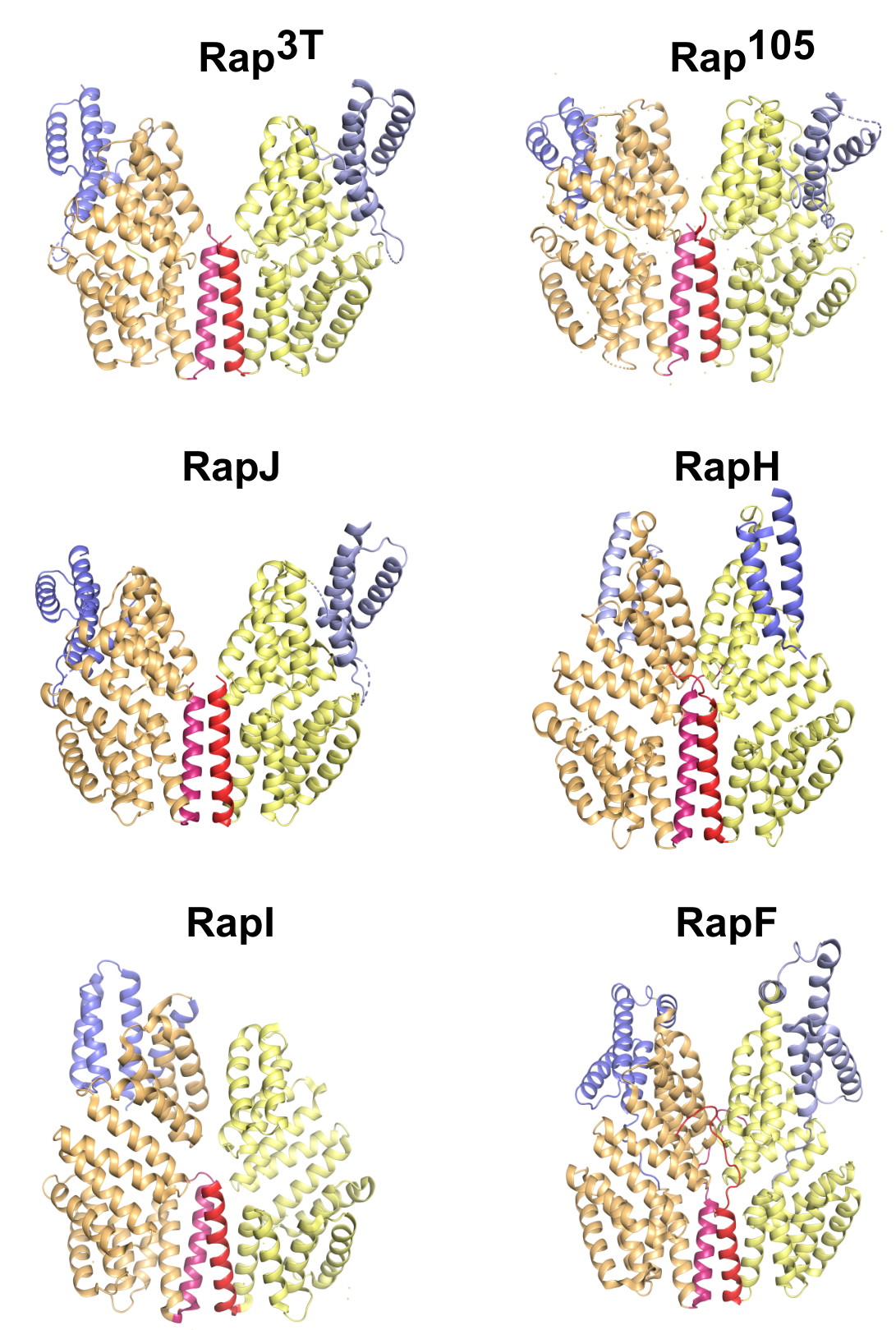

Supplement: S3 Fig — Cartoon representation of the dimers observed in the crystal structures of Rap3T, Rap105, RapJ (PDB 4GYO), RapH (PDB 3Q15), RapI (PDB 4I1A), and RapF (PDB 4I9E). All proteins show a similar dimerization mode interacting mainly with the TPR domains (orange-yellow) mainly through the C-terminal helix (in tones of red) with the 3HB domains (blue tones) facing outward from the dimer. (TIFF) [file pbio.3002744.s003.tiff]

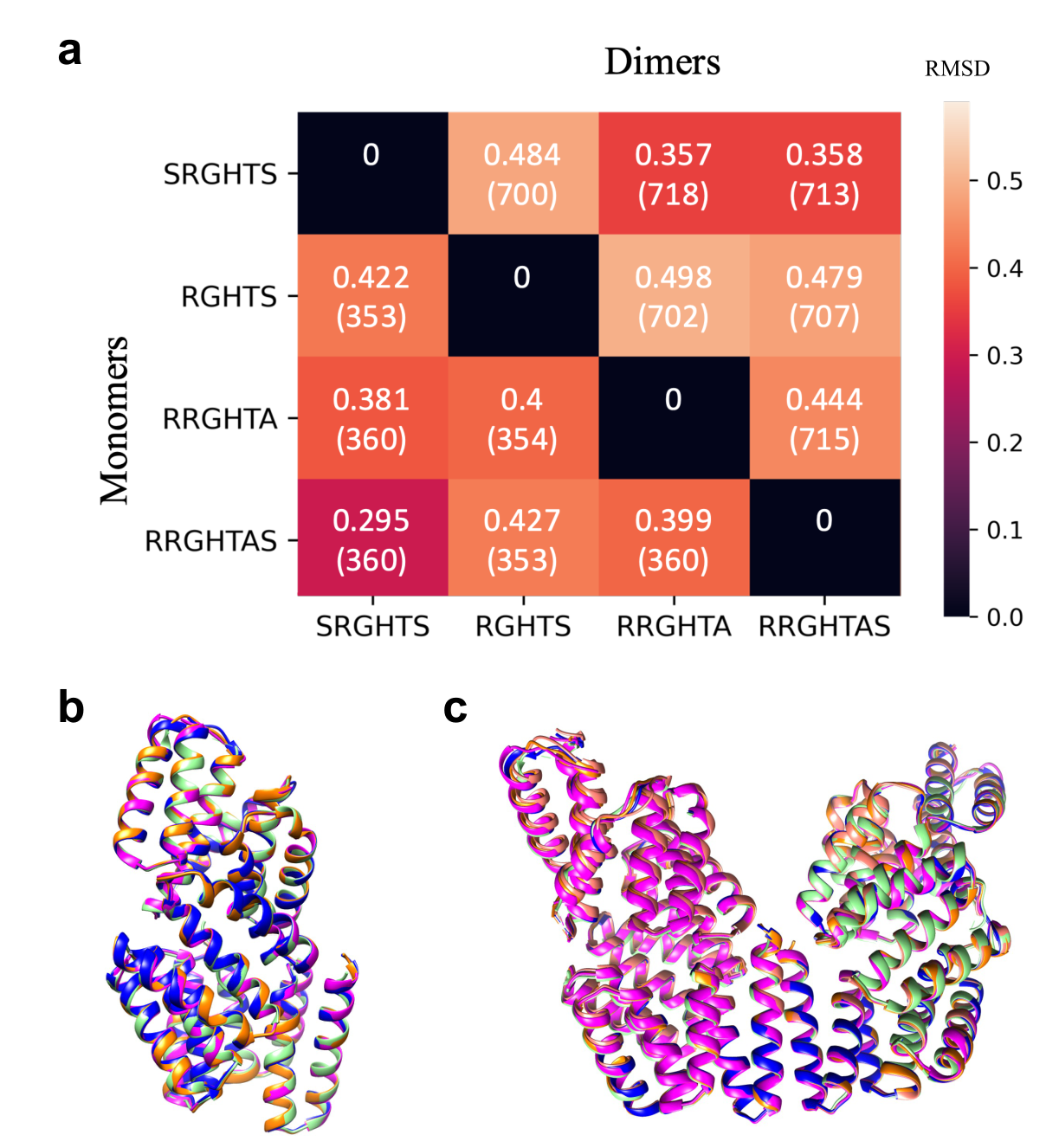

Supplement: S4 Fig — (a) RMSD calculation from the superimposition of Cα atoms of Rap3T structures in complex with peptides SRGHTS, RGHTS, RRGHTA, and RRGHTAS. RMSD were calculated from individual monomers (lower) and dimers (upper). Number of atoms used in RMSD calculation is showed between parenthesis. (b) Superposition of 4 Rap3T-Peptide complexes with different pheromone variants in monomeric state RRGHTAS (blue), RRGHTA (pink), RGHTS (light green), and SRGHTS (orange). (c) Superposition of dimers for the 4 Rap3T complexes with peptides RRGHTAS (blue), RRGHTA (pink), RGHTS (light green), and SRGHTS (orange). (TIFF) [file pbio.3002744.s004.tiff]

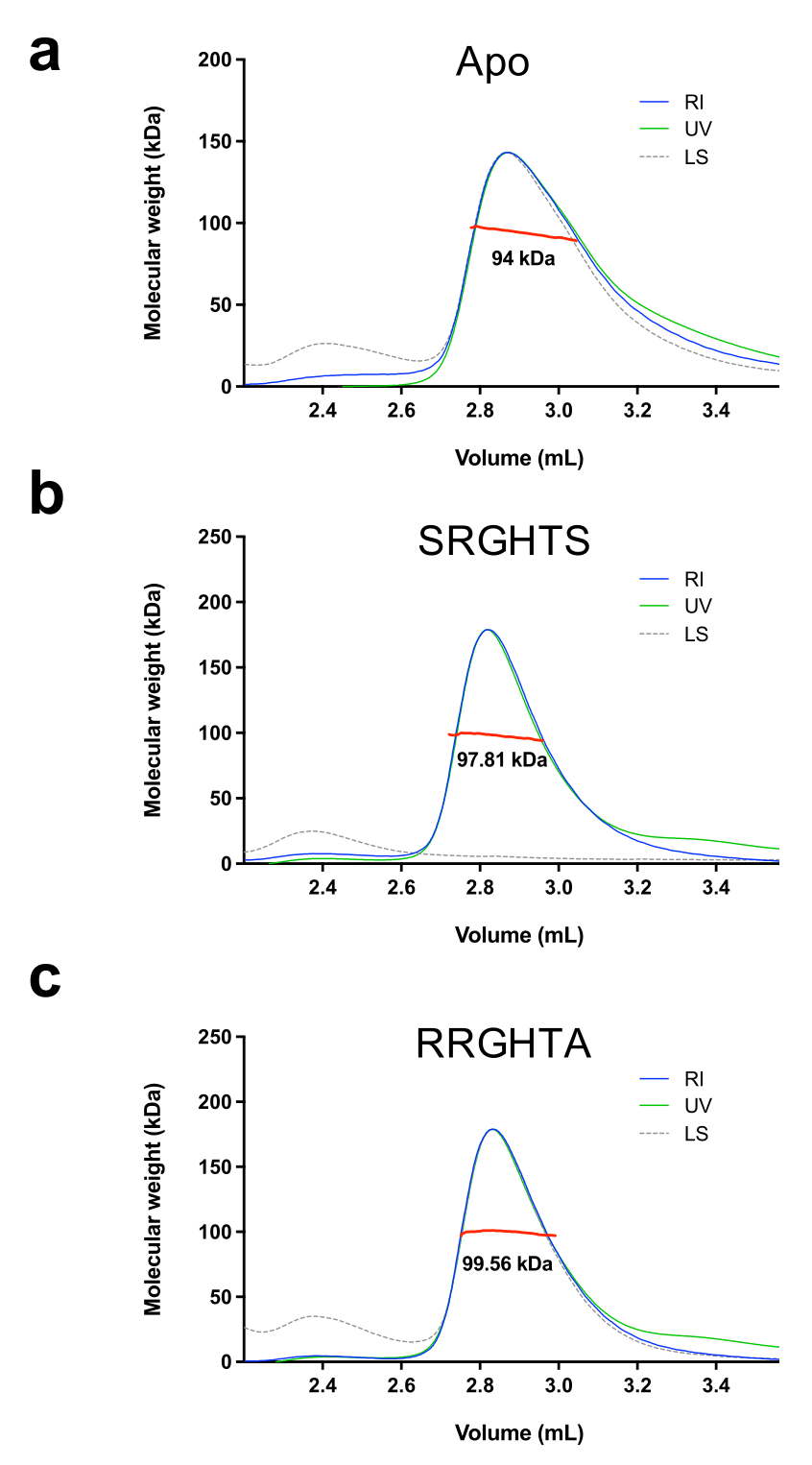

Supplement: S5 Fig — Size exclusion chromatography multi-angle light scattering (SEC-MALS) chromatograms of Rap3T in absence (a) and presence of SRGHTS (b) and RRGHTA (c) pheromones. Chromatograms show the readings from the light scattering (dashed black line), refractive index (blue line), and ultraviolet (green line) detectors. The vertical axis represents the molecular mass. The horizontal red curves represent the calculated molecular masses. In all cases, the molecular weight calculated corresponds to a dimeric form (theoretical MW for the apo, SRGHTS and RRGHTA dimers are 90, 91.2, and 91.3 kDa, respectively). (TIFF) [file pbio.3002744.s005.tiff]

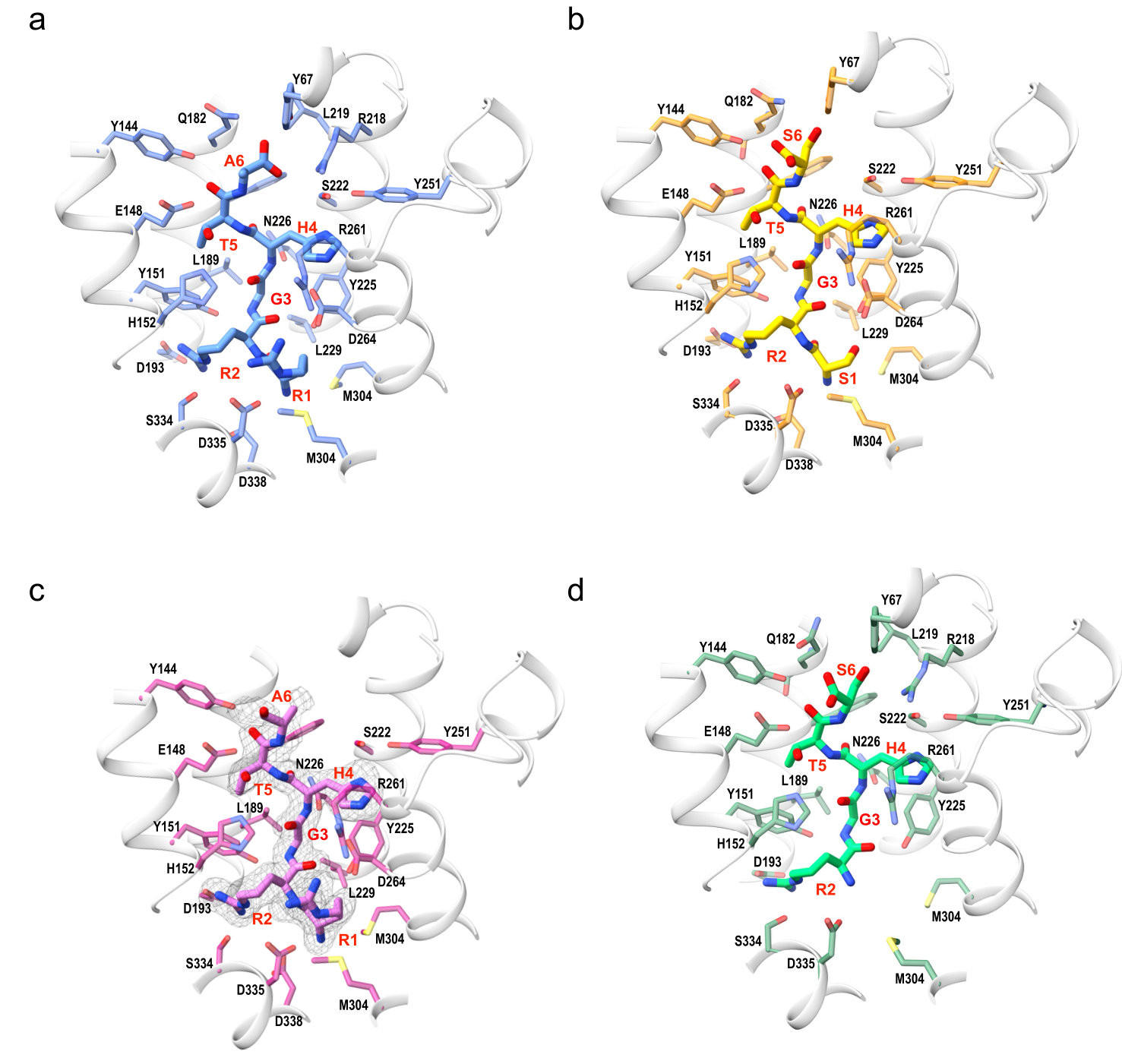

Supplement: S6 Fig — Close view of the peptides (a) RRGHTA, (b) SRGHTS, (c) RRGHTAS, and (d) RGHTS bound to Rap3Tshowing the peptides and the Rap3T interacting residues in sticks. The Rap3T structural elements where the recognition residues are placed are shown in translucent white cartoon. Signaling peptides and Rap3T interacting residues are labeled in red and black, respectively. In b, the Fo − Fc omit electron-density Fourier map contoured at 2σ and carved within 2.5 Å for RRGHTAS is shown in black. (TIFF) [file pbio.3002744.s006.tiff]
