# Supplementary material for: Extracellular proteolysis of tandemly duplicated pheromone propeptides affords additional complexity to bacterial quorum sensing
Source: PLoS Biol. 2024 Aug 13;22(8):e3002744. doi: 10.1371/journal.pbio.3002744 (PMC11343458; doi:10.1371/journal.pbio.3002744)
Supplement: Attachment — Submitted filename: response to reviewers.docx [file pbio.3002744.s021.docx]

**Reviewers´ Comments:**

**Reviewer #1:**

The manuscript "Maturation Mechanisms and Binding Dynamics of Duplicated Peptides Unlocks Extra Layers of Control in Bacterial Quorum Sensing" by Felipe-Ruiz et al. aims to decipher the regulatory role of peptide pheromone duplication in the RRNPPA bacterial communication system.

The authors use two Rap-Phr systems of bacteriophages phi105 and phi3T as models to investigate two types of duplications.They combine in vitro interaction measurements with maturation assays using bacterial strains deleted of distinct protease genes and analyze their results in the light of structural data. They also analyze the conservation of these 2 types of pheromone duplication among other Rap-Phr systems. Overall, the experiments are well described and the results clearly illustrated. The main result is the need, in both systems, for distinct specific proteases to produce the two active mature peptides. As each protease is expressed under specific metabolic conditions, they will produce distinct mature peptides in the case of divergent duplication, or distinct quantity of the same peptide in the case of conserved duplication, and will therefore regulate distinct biological processes (sporulation, biofilm formation..)- Altogether, this multidisciplinary study demonstrates that propeptide maturation plays an essential role in the regulation of these QS systems and that duplication of the mature peptide allows a single system to regulate distinct function depending on the availability of the secreted proteases. In view of the novelty of the results presented here, I recommend this manuscript for publication in PloS Biol.

We thank the reviewer for their support.

However, the discussion concerning the cooperative binding of the peptide in both systems could be improved to support the regulatory mechanisms proposed for the two systems. In particular, the interpretation of the MST experiments and the calculation of the Hill coefficient could be better explained (Sup Fig 1 and Fig 1d).

Following the reviewer indications, we have made a more in-depth discussion regarding the pheromone cooperative binding in both systems in the context of the proposed regulatory mechanisms (page 18, lines 457-41). Also, we have improved the explanation for the interpretation of the MST experiments and the calculation of pheromone binding to the receptors in the Methods and Methods and Figure captions.

The authors consider that the reported cooperativity is in agreement with the dimeric organization of the Rap proteins but several papers (Baker and Neiditch 2011, Parashar et al 2013, Parashar et al 2011) showed that Rap proteins are monomeric in solution and most dimers observed in the crystal structures of Rap proteins are not considered as stable.

We agree with the reviewer that although most Rap structures show a dimeric organization in their crystalline form, which is very similar to that observed for different members of the RRNPPA family, it has been described that some of these Raps would show a monomeric state in solution according to the technique used to evaluate its quaternary organization. In addition, other types of quaternary organization such as tetramer have also been described for Rap proteins.

To clarify the quaternary organization in solution of the Rap proteins analyzed in the present work we have used SEC-MALS. The data presented in the revised version of the manuscript show that Rap^3T^ is dimeric in solution both in its Apo form and in the presence of each of the two mature pheromones. In the case of Rap^105^, the protein is more unstable and shows a high propensity to aggregate, which has prevented adequate characterization in solution. In any case, structural comparison with Rap^3T^ shows a similar dimerization mechanism exploiting identical structural elements for dimer formation, which is also similar to the observed in different crystal structures of Rap proteins, supporting the dimeric organization that would allow the cooperativity observed in the MST analysis.

This information and the experimental results are described in the manuscript text and presented in two new Supplementary Figures (Supplementary Fig. 3 and Supplementary Fig, 5).

In addition, to reinforce the main finding that distinct proteases are required to produce the two mature peptides, further details could be provided to explain the results of the thermal shift assays performed with culture supernatants of bacterial strains lacking each protease. In particular, the significance of the two slopes in Fig. 2c should be better explained and the result would be more convincing with all curves covering the same temperature range. Provide also the results presented in Fig. 2b and 2c for both Imat13T and Imat23T.

The text has been reworded to explain in more detail the 2 slopes observed in Figure 2c, which correspond to those observed in the maturation assays with supernatants of protease-deficient or WT strains, respectively, are indicative of an impaired peptide maturation in these mutants. This observation is discussed in more detail in the subsequent time-course characterization of the Imat1^3T^ maturation for the Δ*wprA* and Δ*bpr* mutants (Figure 2d).

For clarity, the graphs only show the curves covering the temperature range where transitions are observed and which are used to calculate the TM, although all the assays have been carried out in identical temperature ranges. Also, for clarity we have preferred to present 2 alternative representations for the maturation of Imat1^3T^ and Imat2^3T^ peptides. In the case of Imat1^3T^, as in some cases 2 slopes are observed, we have chosen to show the curves and not the TM values in bars. In the case of Imat2^3T^, where there is only one slope, we have opted for the representation in bars. In any case, all the curves from the thermofluor are now shown in the new Supplementary Fig. 2.

Some minor issues should also be considered:

-       Missing references

P4, line 65: add references for the conformational change induced upon peptide binding to RRNPPA

Reference added

P4, lines 66-69: what is described here is only true for the Rap proteins but not for the RRNPPA transcription factors. Modulate the statement.

The reviewer is right and the sentence has been rephrased.

P5, line 102: Briefly introduce the arbitrium QS system of phage phi3T

Arbitrium system in phi3T has been introduced

P6, line 140: Rap dimerization is controversial. Discuss and add references.

Discussed and references has been added

P9, line 215: add a reference for the prototypical Rap dimer

References and one Supplementary Figure have been added.

P16, line 407: add a reference for the neofunctionalization hypothesis

Reference added

- Figures

In Sup Figure 1, explain what the x-axis represents. Dose = peptide concentration ? unit?

It has been clarified indicating that is peptide in Molar concentration.

In Sup Table 1: add the CC1/2 values in the statistics for each data set and the Ramachandran data in the refinement statistics. Add a legend explaining Rmerge, Rwork, Rfree, unit of B-factors

The table has been modified to include the corrections indicated by the reviewer.

The resolution of Figures 2c and 2d should be increased.

Corrected

Explain in the legend of Figure 2d how the relative maturation is calculated.

The explanation has been introduced

Sup Figure 3: Add a panel showing an omit map calculated with the complex of panel a) showing that electron density is missing for the terminal residue of the heptapeptide RRGHTAS. Discuss if there is enough space in the binding pocket to accommodate this additional C-terminal residue.

The omit map has been included in Figure (now Supplementary Figure 6). The presence of additional space in the binding pocket to accommodate the additional residue is indicated in the text.

**Reviewer #2:**

This manuscript very thoroughly investigates the function of duplicated pheromones in quorum sensing (QS) in Bacillus mobile genetic elements.  This study involves the RRNPPA family of receptors and small unmodified peptide QS molecules.  The first studied members of this system were the Rap/Phr that controlled sporulation and genetic competence in B. subtilis. Those Phr encoded a pre-peptide with the mature peptide encoded by the C-terminal pentapeptide.  Since then, many more Rap/Phr sequence have been revealed, and many of the Phr genes encode more than one mature peptide.  This had led to the hypothesis that the duplication was for the purpose of divergence in the function of the peptide.  This study analyzes the production and functions of the duplicated peptides and shows that they have highly conserved function and are not under divergent selection.

I just have one small suggestion which is to indicate how you are defining pseudopeptide on page 16.  Some speculation as to the function of the pseudopeptide based on what you have learned in this study would also be useful.

We consider pseudo-repetition as internal peptide sequence non-identical to the C-terminal pheromone but differing 1 o 2 aminoacids. In several cases, this internal pseudo-repetition can also be observed as C-terminal pheromone for other systems. Therefore, the calling it a pseudo-peptide does not imply at any point a suggestion of lack or reduced activity. we have reformulated the text to clarify this point and to avoid misunderstandings for the reader.

**Reviewer #3:**

The manuscript entitled "Maturation Mechanisms and Binding Dynamics of Duplicated Peptides Unlocks Extra Layers of Control in Bacterial Quorum Sensing" is well-written and rationale presented. It explores the hypothesis that some cell-cell communication (CCC) peptides encode multiple active peptides within their sequence, that these are revealed by extracellular proteases, and in doing so provide an addition level of regulation for CCC. Further, these peptides are part of the widely distributed  RRNPPA superfamily, such that the findings have broad implications.

Using genetic, biophysical and structural data, complemented by comparative genomics, the manuscript reveals a  molecular mechanism for processing of CCC peptides from two Phr pre-peptides. They demonstrate that multiple active peptides are present with the pre-processed forms. They quantify their binding affinities to cognate receptors, capture the structure of the peptides and their receptors, identify processing proteases,  and demonstrate phenotypic consequences of the binding. Thus, the paper is comprehensive in its level of analyses and the conclusion timely and relevant to bacterial population behaviors.

I have only a few minor comments and considerations.

(1)     Please add statistics to 5.

Statistics have been added.

(2)     Figure 2 suggests that the protease WprA is a major contributor to post-export processing of the peptides. However, I don't think the studies included a complemented strain. This would be highly recommended. Related, the addition of Imat13T and Imat23T to the phenotypes tested in Fig 5 is a very good addition. When the interpretation is combined with data presented in figure 2, it suggests that these peptides are processed by endogenous proteases. While not required, it would be rigorous to include the reporters in the multiple protease knockout strain, were the Phr peptide would be predicted to behave as the negative control.

We thank the reviewer for the suggestion to perform the assays with the protease-complemented strain, but consider that the use of multiple immature peptides showing different behavior in the strains validates the point mutants used. Regarding the reporter assays, our primary objective was to confirm *in vivo* propeptide maturation, thereby enhancing the study's relevance. Analyzing the maturation of individual immature pheromones across all protease-deficient strains would require the transfer of the reporter system to nine different strains (the eight individual protease-deficient strains plus WB800), an arduous task which we consider would not provide proportionate additional information.

(3)     The resolution of images 2C& 2D is low.

The figure has been modified to improve the resolution.
